# Assessment methods and the validity and reliability of measurement tools in online objective structured clinical examinations: a systematic scoping review

**DOI:** 10.3352/jeehp.2021.18.11

**Published:** 2021-06-01

**Authors:** Jonathan Zachary Felthun, Silas Taylor, Boaz Shulruf, Digby Wigram Allen

**Affiliations:** 1 School of Medicine, The University of New South Wales, Kensington, NSW, Australia; 2 Office of Medical Education, University of New South Wales, Sydney, NSW, Australia; 3 Centre for Medical and Health Sciences Education, Faculty of Medical and Health Sciences, University of Auckland, Auckland, New Zealand; Hallym University, Korea

**Keywords:** Australia, COVID-19, Objective Structured clinical examination, Online assessment, Educational technology

## Abstract

The coronavirus disease 2019 (COVID-19) pandemic has required educators to adapt the in-person objective structured clinical examination (OSCE) to online settings in order for it to remain a critical component of the multifaceted assessment of a student’s competency. This systematic scoping review aimed to summarize the assessment methods and validity and reliability of the measurement tools used in current online OSCE (hereafter, referred to as teleOSCE) approaches. A comprehensive literature review was undertaken following the Preferred Reporting Items for Systematic Reviews and Meta-Analyses extension for Scoping Reviews guidelines. Articles were eligible if they reported any form of performance assessment, in any field of healthcare, delivered in an online format. Two reviewers independently screened the results and analyzed relevant studies. Eleven articles were included in the analysis. Pre-recorded videos were used in 3 studies, while observations by remote examiners through an online platform were used in 7 studies. Acceptability as perceived by students was reported in 2 studies. This systematic scoping review identified several insights garnered from implementing teleOSCEs, the components transferable from telemedicine, and the need for systemic research to establish the ideal teleOSCE framework. TeleOSCEs may be able to improve the accessibility and reproducibility of clinical assessments and equip students with the requisite skills to effectively practice telemedicine in the future.

## Introduction

### Rationale

The objective structured clinical examination (OSCE) serves as a component of a broader multimodal assessment process that ultimately endeavors to determine whether a student in the health professions can provide safe and effective patient-centered care [1]. Recently, the coronavirus disease 2019 (COVID-19) pandemic has imposed constraints on physical interactions between students and patients due to social distancing, and has necessitated methodological adaptations in education delivery and assessment. Although educators are broadly familiar with the move to online educational delivery platforms, video conferencing technology in particular should be highlighted as a way to achieve the desired objectivity and structure of the OSCE while respecting contemporary demands for infection risk reduction and improved accessibility, with a relatively neutral budget imposition.

Despite an abundance of literature addressing the in-person OSCE, there is a paucity of information on its online counterpart, which we refer to as the teleOSCE. Nevertheless, the adoption of online platforms for telemedicine presents striking similarities to the transition from the in-person OSCE to the teleOSCE; therefore, telemedicine is an invaluable resource when considering teleOSCE format and design. Just as it may be challenging to establish diagnoses that require tactile assessment or diagnostic maneuvers using a telemedicine platform [2], the assessment of physical examinations may be troublesome over a teleOSCE interface and require alternative assessment modalities. A possible way of resolving this dilemma may be that, with the transition to the teleOSCE platform, the assessment of hands-on skills could shift to complementary testing strategies, such as clinical workplace-based assessments. The teleOSCE is not a perfect reflection of the telemedicine “virtual visit”—as such, fortunately, some of telemedicine’s limitations are surmountable. Examination stations can be enriched by the provision of additional fictional information. Additionally, the issues of assessing physical examinations could be overcome by using an assessment configuration wherein the examinee and simulated patient occupy the same room, with the examiner situated remotely. In a broader context, modeling the teleOSCE on telemedicine consultations may additionally prepare students to function more effectively as future clinicians in an environment that encourages humans to work harmoniously with technological innovations to meet growing healthcare demands [3]. Although teleOSCEs may theoretically have many benefits, they must be proven practical before they can be widely adopted.

### Objectives

This article aimed to summarize the various methods of teleOSCE delivery and assessment in the published literature, with a particular focus on determining their validity, reliability, and ultimately, their utility. On the basis of the findings, key attributes of teleOSCEs are highlighted and suggestions are provided for future endeavors in teleOSCE design.

## Methods

### Ethics statement

This was a literature-based study; therefore, neither approval from the institutional review board nor informed consent was required.

### Study design

This was a systematic scoping review, described in accordance with the Preferred Reporting Items for Systematic Reviews and Meta-Analyses extension for Scoping Reviews (PRISMA-ScR) guidelines [4].

### Protocol and registration

An internal review protocol was developed, but was not registered nor published.

### Eligibility criteria

This review included studies of any form of performance assessment, in any field of healthcare, delivered in an online format. The studies were limited to those published in the preceding 10 years, in an effort to focus on the use of contemporary online technology. Articles were excluded if their focus was on using online technology for teaching or learning and if they were not in English. An online format was defined as any use of technology that permitted the student to undertake the assessment in a remote location from either the patient or examiner (e.g., video recordings of patients or telecommunication technology).

### Information sources

PubMed (from 2010 to July 2020), Scopus (from 2010 to July 2020), and PROSPERO (until May 2021) were searched.

### Search

Two reviewers (J.Z.F., D.W.A.) independently conducted a systematic search for studies examining performance assessments in healthcare delivered in an online format. PubMed (from 2010 to July 2020) was searched using the terms (exploded, all subheadings) as follows:

((online[Title/Abstract]) OR (video[Title/Abstract]) OR (remote[Title/Abstract]) OR (web[Title/Abstract])) AND ((OSCE[Title/Abstract]) OR (long case[Title/Abstract]) OR (short case[Title/Abstract]) OR ("performance assessment") OR ("performance examination")) NOT (teaching[Title/Abstract]) NOT (learning[Title/Abstract])

Our search was limited to studies in humans in English and was supplemented by hand-searching the reference lists of the identified papers. Scopus was utilized to search for recent articles citing seminal papers without using a formal search strategy. The PROSPERO database was searched using the above-described strategy (title/abstract portion redacted) to confirm that no recent or ongoing systematic scoping studies had been completed on the topic.

### Selection of sources of evidence

**Fig. 1.**
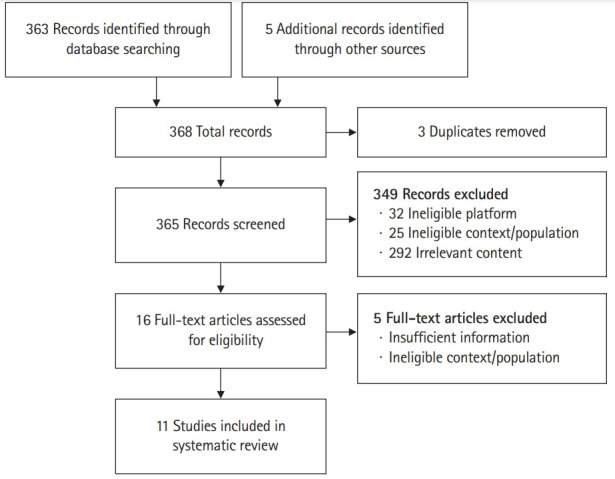
Preferred Reporting Items for Systematic Reviews and Meta-Analyses extension for Scoping Reviews flow diagram demonstrating the article selection process [5].

Two authors (J.Z.F., D.W.A.) screened the titles and abstracts of identified studies based on inclusion and exclusion criteria [5] (Fig. 1). The full texts of the shortlisted studies were analyzed and evaluated independently for eligibility by the same 2 authors (J.Z.F., D.W.A.). In instances of uncertainty (n=3), the other 2 authors (S.T. & B.S.) were consulted to make a decision by consensus.

### Data charting process

The following data were extracted and entered into a standardized form: publication authors, year, study design, and configuration of the online OSCE (Supplement 1).

### Data items

Articles were included if they featured any variable relating to the method of delivery and method of assessment. Reliability, validity, and acceptability were variables of particular interest.

### Critical appraisal of individual sources of evidence

Not done.

### Synthesis of results

The principal investigators performed an analysis to derive key themes represented in the search strategy output. The themes included the configuration of the teleOSCE, the aims and focus of the study, the primary results, and the subsequent conclusions.

## Results

### Selection of sources of evidence

The search strategy yielded 363 published articles, and 5 additional articles were found by screening the reference sections of appropriate articles. After duplicates were removed, 365 articles were screened. The initial title and then abstract screening excluded 349 articles, leaving 16 articles for full-text analysis. Of these, 3 had insufficient information on the analysis of the online OSCE component of the exam, 1 used video recordings to assess features of the traditional OSCE, as opposed to evaluating the online platform, and 1 focused on assessing telemedicine skills rather than using an online platform for assessment. The exclusion of those 5 studies left 11 articles to be included in the qualitative synthesis for the scoping study (Fig. 1).

### Characteristics of the sources of evidence

The included articles originated from several countries (United Kingdom [6], Canada [7], Northern Ireland [8], United States of America [9,10], Bahrain [11], Qatar [12], Germany [13], Philippines [14], and Taiwan [15]) and focused on participants with different levels of experience (medical students [7,8,10-15], emergency medicine residents [16], pediatric trainees [6], anesthesiology residents [9], surgical residents, and qualified surgeons [13]).

### Critical appraisal within sources of evidence

Not done.

### Results of individual sources of evidence

The relevant data from the included studies addressing the review questions are summarized in Supplement 2.

### Synthesis of results

#### Methods of teleOSCE assessment and delivery

Three studies utilized pre-recorded videos of patients or doctor-patient encounters in place of in-person simulated patients amongst traditional OSCE stations [6,8,12]. Another used the consensus between an expert examiner’s appraisal of pre-recorded doctor-patient encounters and that of a student examinee, to evaluate the student’s knowledge of communication skills [11]. The remaining 7 studies evaluated the degree to which it was feasible to conduct assessments in which remote examiners observed students through an online platform. Two of these studies utilized live video feeds of examinee-patient encounters [7,15]. Four studies supported the use of remote examiners through recorded footage of the examinee-patient encounter, often using a real-time, on-site examiner for comparison [9,13,14,16]. One study placed the student, examiner, and patient all in separate rooms [10].

#### Outcome measures

The studies were highly varied in the outcome measures that were reported, all of which are outlined in Supplement 2. However, all achieved success in at least 1 of the factors of reliability, validity, and acceptability. Four studies commented on reliability, with 2 focusing on internal consistency [8,13], 1 on inter-item correlation [6], and 1 on inter-observer reliability [14]. Ten studies commented on validity, all of which evaluated criterion validity by comparing their teleOSCE method to an in-person format [6-10,12-16]. Two studies used construct validity; 1 study evaluated its’ scoring as an indicator of knowledge growth [11], while the other compared students to residents and experts [13].

## Discussion

### Summary of evidence

Beyond the lessons garnered from telemedicine, this scoping review reveals a developing body of literature outlining attempts at implementing teleOSCEs. Given the inherent differences in the application of telemedicine and OSCE consultations, the findings of this study are imperative for understanding how an online platform may affect the assessment process and outcomes. All the studies retrieved from the literature search reported desirable outcomes for validity, reliability, and/or acceptability regarding the technological innovations analyzed in their methods. While this trend may reflect publication bias to a certain extent, as few studies suggested possible improvements to their methods, these findings nonetheless demonstrate that with careful consideration, coupled with appropriate tailoring to the individual setting, teleOSCEs can achieve the same values that their in-person counterparts aim to attain. Nevertheless, the validity of OSCEs can vary according to the context in which they are performed [17]. More meaningful insights for future studies could potentially be gleaned by evaluating the online assessment process, as opposed to measuring the psychometric outcomes, with a focus on how the online platform impacts students’ performance and examiners’ judgments.

For example, it is critical to understand whether substituting an examiner with a camera has an impact on students’ performance. The audience effect is a component of social facilitation theory that attempts to explain performance changes in the presence, or perceived presence, of others [15]. Simply put, an individual’s performance of unfamiliar and complex tasks is impaired in the presence of others, while the presence of others improves the performance of tasks that have been mastered [15]. Hamilton and Lind [18] suggested that performing a recorded examination may replicate the audience present when performing in front of an examiner in close proximity. To optimize the OSCE pre-exam process, technological advancements, including e-learning orientation modules and eye-tracking enriched training videos, have been utilized to improve examiners’ and examinees’ preparation for OSCEs, respectively [19,20]. However, as shown by this review, there have been minimal practical investigations of these technologies in high-stakes examinations.

Additionally, the review revealed little about whether examiners extract different information about student performance from teleOSCEs and in-person assessments. Traditionally, 1 or more examiners in close proximity, in addition to a patient and an examinee, occupy the room, and the examiners’ observations usually comprise the majority of the assessment [15]. The examiners are often free to move around the room, altering their perspective and interactions with the examinee. This possibility is more limited and contingent on available resources within a teleOSCE. For instance, Chen et al. [15] implemented a camera that could pan 360°, theoretically allowing examiners to obtain more information than is possible using a stationary camera. Furthermore, the use of 2 cameras might enable an isolated examiner to evaluate multiple perspectives simultaneously, which cannot be replicated for an in-person examiner. With regard to the set assessment task, Chan et al. [7] suggested that a single camera is adequate for history-based stations, while physical examination–based stations require a second camera. This scoping review has demonstrated a consistently good correlation between the assessment of recorded OSCE stations and live in-person examinations, but is lacking in guidance as to how a camera may limit—or expand—the ability of examiners to observe students as they perform the examinations.

The influence of an online platform on the derivation of emotional and perceptual information by simulated patients, examiners, and examinees is of paramount interest and largely unexplored in the studies analyzed herein. Cognitive theories assert that such perceptions are the composite of interrelated cues from a range of sources, including facial expressions, body language, and contextual information, all integrated through the construct of an individual’s knowledge, beliefs, biases, gender, ethnicity, level of experience, and emotional state [21-23]. Hence, restricting the input to what can be garnered from a screen may impede examiners’ capacity to make these judgements. For instance, if a close-up shot restricts the frame to the face of an examinee, an examiner could miss the fidgeting of hands or tapping of feet, which may represent important information for gauging an individual’s confidence, poise, and capability. This may explain why Chan et al. [7] and Chen et al. [15] demonstrated differences in results between on-site and remote examiners, but only when using the more subjective global rating scale. Conversely, research suggests that humans are extraordinarily well-adapted to perceiving emotional states, with the ability to derive conclusions about complex emotions from photographs of human faces in just 1 second [24]. The ability of an individual to exercise this cognitive skill across a range of clinical domains, such as mental health assessments and the delivery of bad news, is a vital component of operating as a competent practitioner. As such, it is important to consider how the configuration of a teleOSCE may influence this process and whether multiple camera angles are necessary to capture detailed contextual data, or if sufficient information can instead be gleaned from a more minimalist approach.

### Limitations

An important limitation of this article is that it explored methods of teleOSCE delivery and assessment in the published literature. It is possible that education providers may be conducting teleOSCEs without publishing their findings; as such, the conclusions of this analysis may have been influenced by positive publication bias. Furthermore, only manuscripts published in English were reviewed and cost outcomes were not reported. Lastly, the methodological quality of several studies could have been enhanced by including an in-person OSCE control group for comparison.

### Suggestion

The heterogeneous approach to teleOSCE structure and inconsistencies in the evaluation of the psychometric aspects of online assessments have contributed to the lack of consensus surrounding an appropriate teleOSCE configuration. This is largely due to the small sample size of published studies that can furnish the basis for evaluating teleOSCE delivery and assessment. As such, future empirical research is necessary to establish the ideal format for teleOSCE assessments. We suggest that future studies aim to compare in-person assessments with teleOSCEs using matched cohorts and employ established measures of reliability and validity to present their results. Moreover, additional research and—arguably more importantly—increasingly innovative ideas are necessary to adapt assessments of physical examinations to online platforms. The probable future shift to teleOSCEs may necessitate that certain aspects of performance assessment be undertaken in other formats such as clinical workplace assessments.

## Conclusion

There are many examples of successful teleOSCE delivery and assessment that have achieved favorable results in terms of reliability, validity, and acceptability for students and examiners. The video interface is most suited to clinical scenarios that rely on communication skills and observations as opposed to physical examinations. For more complex observation tasks, it may be useful to employ multiple cameras and fabricated clinical information that moves beyond what is possible to assess using current technology, such as the provision of vital signs, physical examination findings, or investigation results. Alongside this guidance and insights that will be gleaned from future studies, the broader adoption of teleOSCEs will be possible. This may foreseeably improve the accessibility and reproducibility of clinical assessments whilst contributing to equipping students with an increased capacity to subsequently undertake online patient assessments as future clinicians.

## Supplementary materials

Supplementary files are available from Harvard Dataverse: https://doi.org/10.7910/DVN/28ZNMI

Supplement 1. Data extraction form.

Supplement 2. Characteristics of key findings of included studies.

Supplement 3. Audio recording of the abstract.
